# Mechanism of erosion of nanostructured porous silicon drug carriers in neoplastic tissues

**DOI:** 10.1038/ncomms7208

**Published:** 2015-02-11

**Authors:** Adi Tzur-Balter, Zohar Shatsberg, Margarita Beckerman, Ester Segal, Natalie Artzi

**Affiliations:** 1 The Inter-Departmental Program of Biotechnology, Technion—Israel Institute of Technology, Haifa 32000, Israel; 2 Institute for Medical Engineering and Science, Massachusetts Institute of Technology, Cambridge, Massachusetts 02139, USA; 3 Department of Biotechnology and Food Engineering, Technion—Israel Institute of Technology, Haifa 32000, Israel; 4 Russell Berrie Nanotechnology Institute, Technion—Israel Institute of Technology, Haifa 32000, Israel; 5 Department of Anesthesiology, Brigham and Women’s Hospital, Harvard Medical School, Boston 02115, USA

## Abstract

Nanostructured porous silicon (PSi) is emerging as a promising platform for drug delivery owing to its biocompatibility, degradability and high surface area available for drug loading. The ability to control PSi structure, size and porosity enables programming its *in vivo* retention, providing tight control over embedded drug release kinetics. In this work, the relationship between the *in vitro* and *in vivo* degradation of PSi under (pre)clinically relevant conditions, using breast cancer mouse model, is defined. We show that PSi undergoes enhanced degradation in diseased environment compared with healthy state, owing to the upregulation of reactive oxygen species (ROS) in the tumour vicinity that oxidize the silicon scaffold and catalyse its degradation. We further show that PSi degradation *in vitro* and *in vivo* correlates in healthy and diseased states when ROS-free or ROS-containing media are used, respectively. Our work demonstrates that understanding the governing mechanisms associated with specific tissue microenvironment permits predictive material performance.

## Abstract (web summary)

The degradation of materials used in biological applications has an important bearing on their long term performance. Here, the authors show how porous silicon nanoparticle degradation can be accelerated *in vivo* through the influence of local tissue pathology, likely influencing drug delivery performance.

## Graphical abstract

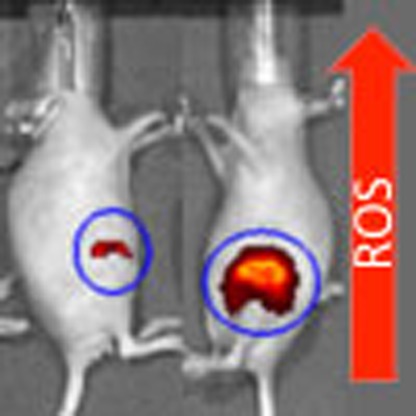

The benefits of localized delivery of therapeutic agents serve as a driving force for the design and synthesis of biomaterials for medical applications^1^,^2^,^3^. The demands from these materials are high, as they must be biocompatible and exert desired therapeutic effect by controlling their structure, morphology and physicochemical properties. Degradable biomaterials that perform their function and are then eliminated from the body are of high interest as permanent residence time of devices and chronic inflammation were found to correlate^4^,^5^,^6^,^7^,^8^. Erosive materials are dynamic; they change shape, morphology and structure in the same time frame they exert their desired effects. Furthermore, when considering the release of embedded drugs, any change in material fate as a result of specific microenvironmental impositions would affect not only material performance and potentially tissue response, but also drug-release kinetics. We have shown that material degradation depends on formulation, shape and implantation site^9^. As biodegradability and biocompatibility are contextual and not a constitutive property of the material, they can only be determined within specific environments. This then needs to be put in the perspective of the specific clinical scenario and the environment the material will be exposed to. Neoplastic states are one example in which a range of modifications including extracellular matrix content, immune cells, enzymes and reactive oxygen species (ROS) may modify device fate. Thus, comparison between material erosion *in vivo* in healthy and diseased states is crucial for elucidating the importance of the physiological microenvironment in determining device performance, tissue response and eventually clinical outcome. This will then help isolating and identifying the precise *in vitro* conditions that represent the most critical parameters determining material behaviour *in vivo*.

Nanostructured porous silicon (PSi) is a promising and versatile material for biomedical applications^10^. The high tunability of PSi-based scaffolds, imparted by the ability to tailor the porosity and surface chemistry^11^,^12^,^13^, together with the biocompatibility and degradability into nontoxic orthosilicic acid^14^,^15^, has already led to the use of PSi as optical biosensors^16^,^17^,^18^, for biomolecular screening^19^, tissue engineering^20^,^21^,^22^ and drug delivery^11^,^12^,^23^,^24^,^25^,^26^,^27^,^28^,^29^, specifically in cancer therapy^12^,^26^,^30^,^31^,^32^,^33^,^34^,^35^. Although PSi emerges as a promising vehicle for local drug release, its *in vivo* fate was not yet studied, and to the best of our knowledge there is no mechanistic study that examines the potential impact of tissue microenvironment on PSi degradation. We hypothesized that pathological state, such as breast cancer, would alter PSi fate *in vivo*. As material degradation will determine drug-release kinetics, the implications are that the tumour state will be directly regulated by material erosion kinetics.

In this study, we examine the effect of local pathology on PSi fate using human breast cancer xenograft model and find that PSi erosion is highly catalysed in the cancerous environment compared with the healthy state owing to the upregulation of ROS. We exploit a noninvasive imaging technique to monitor the fate of fluorescently labelled PSi microparticles *in vivo* in healthy and diseased environments. The fluorescence of the reporter is quenched by the PSi to an extent dependent on the state of oxidation and extent of degradation. This phenomenon by itself is indicative of the differences between healthy and diseased environments, and in particular the impact of oxidative stress on implanted materials. Owing to the modification of the reporter by the environment, we generated a formula that converts fluorescence to mass under artificial *in vitro* conditions that mimic the *in vivo* environment. The formula is created by tracking *in vitro* material fate using inductively coupled plasma atomic emission spectroscopy (ICP-AES) and fluorescence simultaneously, and is then used to convert the *in vivo* fluorescence to mass. We then show that correlation between *in vitro* and *in vivo* material erosion persists only under *in vitro* clinically relevant conditions that capture the main factors determining material fate *in vivo*. The ability to determine and predict material fate *in vivo* under specific environments is the next step in material design that would lead to faster and successful clinical translation.

## Results

### Tracking PSi erosion *in vivo*

Real-time monitoring of material erosion is a key factor in designing and programming the performance of erodible biomaterials. However, traditional determination of *in vitro* erosion cannot always predict *in vivo* performance because of the significant differences in environmental forces *in vitro* and *in vivo*. As such, loss of material integrity, structure and eventually mass may differ dramatically, affecting *in vivo* biomaterial performance. Specifically, the performance of nanostructured PSi-based drug delivery systems is critically dependent on the degradation behaviour of the Si scaffold^11^,^33^,^36^,^37^,^38^.

We studied the degradation profile of nanostructured PSi thin films that were fabricated by anodization process at a constant current density of 15 mA cm^−2^ for 225 s. The etching conditions were adjusted to yield an ~2,500-nm-thick porous layer (porosity of 65% and surface area of ~450 m^2 ^g^−1^) with a typical morphology of interconnecting cylindrical pores. Following the electrochemical etching, the resulting porous films were lifted off from the bulk Si substrate, by applying an electropolishing current, after which the freestanding films were fractured into micron-size particles (2–18 μm) by ultrasonication (see Supplementary Fig. 1a,b).

Most studies have investigated the behaviour of these carriers *in vitro*; however, future clinical applications of these nanomaterials would require characterizing material *in vivo* erosion and establishing clinically relevant *in vitro* conditions under which one can attain similar behaviour in the two domains. Monitoring PSi degradation noninvasively and continuously is challenging. Gu *et al*.^39^ have studied the *in vitro* degradation and dissolution process of luminescent PSi particles and suggested that material fate can be monitored through its intrinsic luminescence. We found that the luminescent signal of the particles is not sufficient post implantation, thus requiring another method for *in vivo* tracking to be employed (Supplementary Fig. 2).

To allow optical monitoring of the *in vivo* degradation process, PSi microparticles were chemically modified by thermal hydrosilylation of undecylenic acid^26^,^38^,^40^,^41^ to enable covalent attachment of Texas-red hydrazide (TRH) dye molecules to the Si scaffold, as illustrated in Supplementary Fig. 1c. Attenuated total reflectance Fourier-transform infrared (ATR-FTIR) spectroscopy was used to confirm the chemical modification of the Si scaffold following thermal hydrosilylation and fluorophore tagging (Supplementary Fig. 1c).

**Figure 1:**
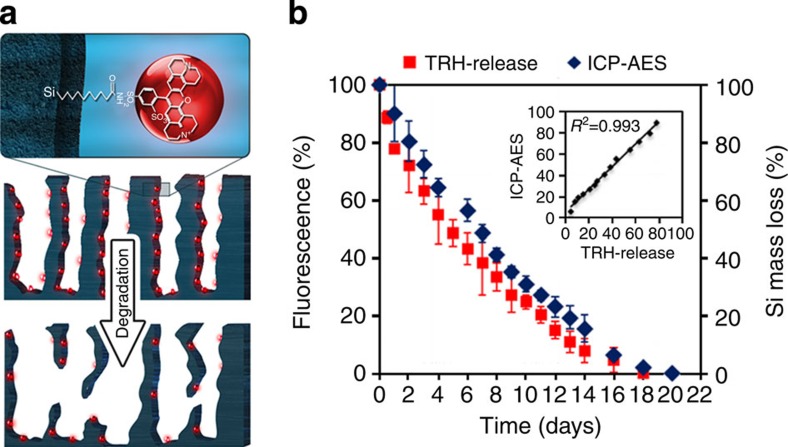
Degradation of TRH-labelled PSi microparticles. (**a**) Schematics of the degradation process of TRH-labelled PSi microparticles and (**b**) *in vitro* Si degradation profiles measured by ICP-AES (blue) and TRH fluorophore release (red). Data are the average percentage±s.d of three independent experiments. Note: the porous nanostructure is characterized by a morphology of interconnecting cylindrical pores that is not reflected in the schematic representation of the PSi.

Figure 1a schematically illustrates the erosion process of the TRH-labelled PSi microparticles. Many studies have shown that the degradation of PSi in physiological media involves the oxidation of the Si scaffold into Si-dioxide, followed by the hydrolysis of the Si–O bonds to release soluble orthosilicic acid species^11^,^14^,^37^,^38^,^42^. In the case of TRH-labelled PSi, as the fluorophore molecules are covalently attached to the Si backbone, their release into the surrounding media can occur only through the degradation of the crystalline Si scaffold. Thus, Si erosion can be monitored by measuring the fluorescence intensity of the aqueous media, into which the Si–TRH species were released, as corroborated by the analytical quantification of the content of Si in the release media using ICP-AES, for TRH-labelled PSi microparticles (Fig. 1b). This assay is commonly used to provide the absolute values of mass changes in the Si matrix and is considered the gold standard method for characterizing PSi degradation^32^,^33^,^36^,^43^,^44^. The ICP-AES and florescence analyses highly correlate (*R*^2^=0.993, Fig. 1b inset) and display a gradual decrease in the Si mass loss, resulting in a complete degradation of the microparticles within ~20 days. These results reveal that fluorescence tracking enables sensitive monitoring of PSi degradation process.

### The effect of the tumour microenvironment on PSi erosion

**Figure 2:**
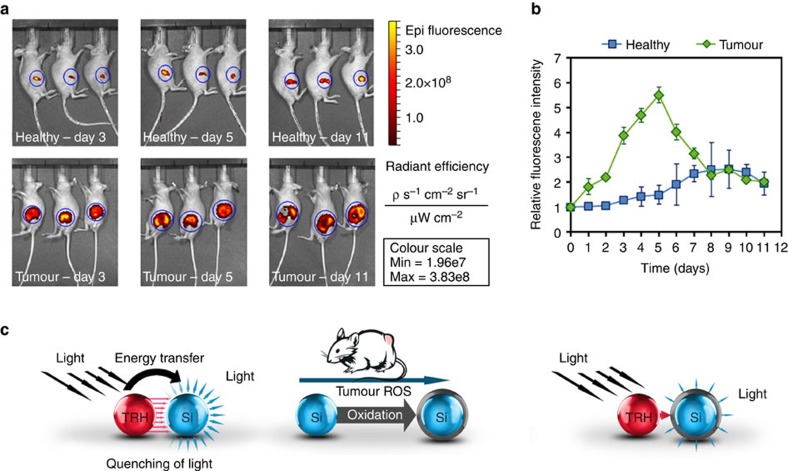
Fluorescence intensity of TRH-labelled PSi particles in healthy and tumour-bearing mice. (**a**) Representative measurements using the *in vivo* imaging system technique and (**b**) analysis of the results. (**c**) Schematics of fluorophore signal in the presence of tumour ROS. Data are the average percentage±s.d of two independent experiments, each including 15 severe combined immunodeficiency (SCID) female mice (10 mice were induced with breast cancer and 5 served as a healthy control).

In order to provide insight into the effect of the physiological microenvironment and disease state on the erosion of the PSi carriers, material mass loss *in vivo* was monitored by intravital tracking of the fluorescently tagged microparticles. Breast cancer tumours were induced by injecting luciferase-expressing human breast carcinoma cells (luc-MDA-MB-231) into the left mammary fat pad of severe combined immunodeficiency mice. These cells were engineered to express luciferase, thus enabling noninvasive imaging of tumour state by quantifying the bioluminescent signal using the *in vivo* imaging system, see Supplementary Fig. 3. Following tumour induction, intratumoral injections of TRH-PSi microparticles (aliquots of 2 mg of particles in 30 μl) were administered and intravital tracking was carried out to visualize and quantify the fluorescence of the labelled particles for 11 days. Similar doses of particles were injected into mammary tissues of healthy mice and monitored in parallel. Distinct differences between Si degradation in healthy and diseased mice are evident, revealing the profound effect of the physiological microenvironments on PSi erosion (Fig. 2a). Interestingly, while one expects to see a decrease in fluorescence as a result of degradation, an increase in fluorescence is observed at early time points. TRH-PSi injected intratumorally display significantly higher fluorescent intensity compared with particles implanted into healthy mammary tissue throughout the timescale of the experiments. Intratumorally injected particles show rapid increase in fluorescence intensity reaching a maximum value on day 5 (5.5-fold increase), followed by a fast decrease, in contrast to a more attenuated response in healthy state (2.5-fold increase by day 9, Fig. 2b).

Similar behaviour of labelled PSi was recently demonstrated in ref. 38 *in vitro*. It was found that the fluorescence intensity of dye molecules attached to the surface and inner pore walls of mesoporous Si particles depends on the oxidation level of the Si scaffold. When fluorescent dye molecules are attached to freshly etched PSi, low levels of fluorescence are observed because of quenching of the emitted light by the highly dense electrons in the PSi semiconductor nanostructure^45^,^46^,^47^,^48^,^49^. As the PSi nanostructure oxidizes, the growing oxide layer facilitates physical separation between the dye donor and the semiconductor acceptor, thus eliminating the quenching phenomenon. This process attenuates energy transfer and results in higher fluorescence emission^38^,^50^.

We hypothesized that the observed differences in the fluorescence intensity of TRH-PSi microparticles injected into healthy and cancerous tissues can be ascribed to the profound differences between the physiological microenvironments as related to ROS concentration and the resulting oxidative stress^51^. We confirmed the elevation in ROS concentration in the tumour microenvironment using ROS-sensitive fluorophore in healthy and cancerous tissues. This fluorophore is optically silent until the attached quenching molecule is cleaved by interacting with an oxidant like ROS, permitting fluorescence emission. High signal is observed in the tumour region, indicative of ROS upregulation, compared with the relatively low signal detected in healthy mice (see Supplementary Fig. 4). The pronounced elevation in ROS concentration in the tumour microenvironment^52^ may induce a faster build up of the Si oxide layer compared with the marginal and sustained oxidation in healthy state. Hence, in the diseased environment the quenching phenomenon is eliminated, enabling tracking the changes in fluorescence signal as the PSi degrades, as schematically illustrated in Fig. 2c.

### Identifying physiological conditions defining *in vivo* behaviour

**Figure 3:**
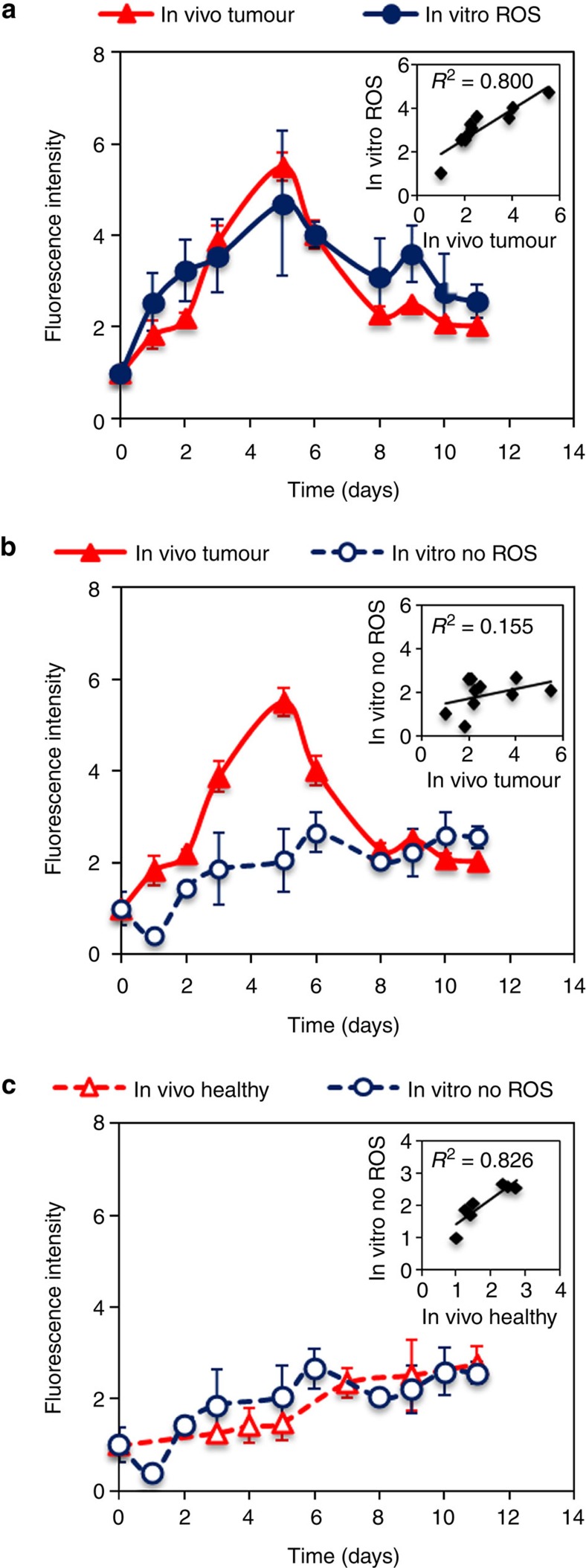
*In vivo* and *in vitro* measurements of the fluorescence intensity of TRH-labelled PSi particles. (**a**) The fluorescence intensity of particles intratumorally injected in comparison with the intensity measured *in vitro* in the presence of ROS (*R*^2^=0.796) and (**b**) *in vitro* without ROS (*R*^2^=0.076). (**c**) The fluorescence intensity of particles injected into the healthy mammary fat pad in comparison with the intensity measured *in vitro* without ROS (*R*^2^=0.860). Data are the average percentage±s.d of two independent experiments, each including 15 SCID female mice (10 mice were induced with breast cancer and 5 served as a healthy control).

We hypothesized that the *in vivo* erosion of PSi in healthy and tumour environments, as reported by the fluorophore, is a result of two mechanisms: the actual degradation of the Si scaffold and also the modification of the reporter’s signal by the build up of the oxide layer that further modifies the degradation^53^. In order to elucidate the contribution of each mechanism, we constructed artificial conditions *in vitro* that allow for controlled addition of physiologically relevant ROS concentrations to examine the impact of ROS on PSi degradation. Once able to eliminate the effect of the environment on the fluorophore signal, we will be able to convert fluorescence signal to mass loss. However, as the cancerous microenvironment presents additional disparities beyond ROS upregulation compared with the healthy state, we examined the impact of other possible factors on PSi degradation *in vitro*, including acidic pH (6.5 compared with 7.4) and addition of proteins whose adsorption to PSi particles following their *in vivo* administration may alter the PSi degradation profile. The degradation was monitored *in vitro* using PBS or human serum, at pH 6.5 or 7.4, with or without ROS present and their combinations (see Supplementary Fig. 5). PSi degradation profile under each of these conditions was similar, unless ROS was used, the latter leading to significant acceleration in degradation. These experiments support our hypothesis that the determinant factor accelerating PSi erosion in the tumour microenvironment is ROS upregulation. Specifically, 3-morpholinosydnonimine N-ethylcarbamide (SIN-1) was used to generate physiologically relevant levels of peroxynitrite (OONO^−^; refs 38, 54, 55), a highly reactive oxygen species involved in human carcinogenesis^56^. Figure 3 summarizes the *in vitro* effect of PBS with and without peroxynitrite on the fluorescent intensity of TRH-PSi microparticles in comparison with their *in vivo* behaviour, observed in healthy and cancerous tissues. The initial increase in fluorescence intensity of the particles by a factor of 4.7 up to day 5 (Fig. 3a) followed by a fast signal reduction is recapitulated *in vitro* by adding the oxidizing agent OONO^−^ to the degradation media. A linear correlation between the fluorescence signal *in vitro* and *in vivo* (*R*^2^=0.796) is attained under these conditions. Control *in vitro* experiments with PBS show a marginal increase in fluorescence (Fig. 3b) and poor correlation with *in vivo* behaviour in cancerous tissue (*R*^2^=0.076). However, this *in vitro* behaviour (in the presence of PBS) correlates with *in vivo* fluorescence when particles are injected into healthy tissue with low oxidation levels (Fig. 3c, *R*^2^=0.860), thus corroborating our hypothesis that oxidative stress is a key factor determining PSi *in vivo* erosion. These results reveal that traditional *in vitro* assessment of particles’ fluorescence using PBS buffer is not indicative of *in vivo* behaviour in diseased tissues, emphasizing the importance of developing preclinically relevant *in vitro* conditions under which one can attain similar behaviour *in vitro* and *in vivo*.

### Generating a formula to convert fluorescence to mass loss

**Figure 4:**
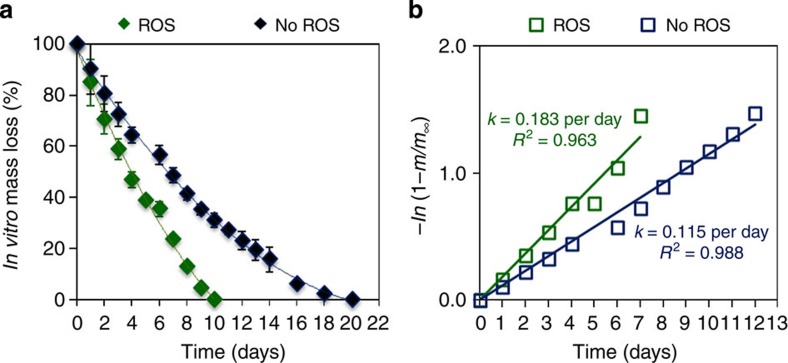
*In vitro* Si mass loss kinetics of TRH-PSi microparticles under physiologically relevant levels of ROS. (**a**) *In vitro* Si mass loss profiles measured by ICP-AES in PBS buffers with ROS (green) and without ROS (blue) and (**b**) corresponding calculated erosion rates. Data are the average percentage±s.d of three independent experiments. Both environments show a logarithmic degradation (that is, increase in Si content in the aqueous environment), with a rate of *k*=0.183 per day for ROS solution and *k*=0.115 per day for PBS buffer.

While the presence of ROS modifies the signal of our reporting molecule, it is also expected to enhance the degradation of PSi, as PSi degradation in aqueous solutions involves oxidation of Si to Si-dioxide, followed by the hydrolysis of Si–O bonds to release orthosilicic acid^38^. Thus, elevated levels of ROS will accelerate the degradation of intratumorally injected particles, resulting in signal reduction. To isolate the autocatalytic effect of enhanced degradation with amplified oxidation while excluding the effect on the fluorescent reporter molecule, *in vitro* Si mass loss of TRH-PSi microparticles was quantified using the ICP-AES analysis under physiologically relevant levels of peroxynitrite (Fig. 4a). While this method is vastly used to accurately monitor Si erosion *in vitro* and *in vivo*^12^,^32^,^44^,^57^,^58^, it requires killing animals at each time point of the experiment in order to determine the Si content in the tissue. However, this method allows us to compare *in vitro* erosion under artificial conditions without ‘contaminating’ the profile with other parameters that do affect the fluorescent reporter. Indeed, Si erosion measured with ICP-AES is enhanced in the presence of ROS, resulting in complete degradation of the microparticles within 10 days. In comparison, the erosion profile in PBS depicts a gradual degradation, lasting for ~20 days (Fig. 4a). This finding corroborates that the acceleration of Si degradation is a result of oxidative stress, and not a simple modification of the reporters’ signal. Si mass is presented on a modified log-linear scale as the erosion profiles fit exponential decay^36^ (Fig. 4b). A linear fit to the data reveals enhanced erosion rate in the presence of physiologically relevant levels of peroxynitrite compared with PBS only, with erosion rate constants of *k*=0.183 and 0.115 per day for the OONO^−^ solution and PBS, respectively.

### Calculating Si *in vivo* erosion

**Figure 5:**
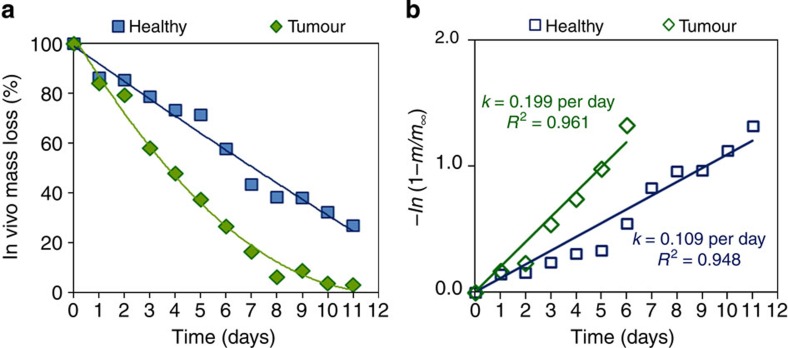
*In vivo* Si mass loss and erosion profiles. (**a**) *In vivo* Si mass loss profiles and (**b**) erosion rates calculated for TRH-labelled PSi microparticles in healthy and tumour environments.

The excellent correlations between PSi *in vitro* and *in vivo* fluorescence, both in healthy and diseased states (Fig. 3), suggest that we can use the conversion between fluorescence and mass loss generated *in vitro* (see Supplementary Fig. 6) to convert Si *in vivo* mass loss from *in vivo* fluorescence. Figure 5a presents *in vivo* Si mass loss calculated for the labelled microparticles in healthy and tumour environments. The PSi *in vivo* erosion rate is significantly enhanced in the tumour environment compared with the healthy state, with *k*=0.209 and *k*=0.112 per day (Fig. 5b), respectively. Establishing these correlations allows for the assessment of *in vivo* Si mass loss profiles while eliminating the need to kill animals at each time point of the experiments, presenting a generic methodology to infer device performance noninvasively and directly from the observed *in vivo* fluorescence. It is worth mentioning that PSi *in vivo* degradation kinetics can be tuned by changing particle size and porosity, but will be further modified as a function of the injection dose/site and tumour type. Hence, our approach of examining PSi microparticles in light of their specific clinical application and the environment they would be exposed to should be employed to unravel the impact of the physiological microenvironment on material performance.

In summary, this work demonstrates that material performance is contextual and hence should be studied in light of the intended clinical use. PSi has emerged as a material with high clinical potential as its high surface area provides ample opportunities for loading drugs and molecules for therapeutic benefits. Although PSi degradation largely determines the release of embedded drugs, its erosion profile under pathological states that are of clinical relevance was not studied. Understanding the factors affecting PSi erosion *in vivo* would facilitate its rapid development and clinical usage as a drug carrier. We now show that PSi erosion is enhanced in the tumour microenvironment compared with the healthy state as a result of elevation in oxidative stress, see Fig. 2. The relationship between fluorescence intensity used to monitor PSi *in vivo* erosion and Si mass with time is complex and is being affected by ROS content in the Si milieu, as we show in Fig. 3. Using ICP-AES as a method to track *in vitro* Si degradation together with optical imaging used to monitor *in vitro* and *in vivo* Si fluorescence, we were able to convert the complex fluorescent signal into material mass. This approach enables noninvasive and continuous tracking of PSi erosion with time. PSi erosion almost doubled in the cancerous tissue, highlighting the need to examine materials in light of their clinical application (Fig. 5). This approach should be applied to any material and clinical scenario to attain predictive performance. Specific clinical scenarios may require further material optimization but would facilitate successful clinical outcome and translation of new materials to the clinic.

## Methods

### Fabrication and characterization of porous Si carriers

*Fabrication of PSi microparticles*. Mesoporous Si films are fabricated from single-side polished p+ <100> silicon wafers (~1 mΩ-cm, B-doped, from Siltronix Corp, France) using an electrochemical etch process, in a 3:1 (v/v) solution of aqueous HF (48%, Merck, Germany) and ethanol (99.9%, Merck), at a constant current density of 15 mA cm^−2^ for 225 s. Si wafers with an exposed area of 6.16 cm^2^ are contacted on the backside with an aluminium foil and mounted in a Teflon-etching cell; a platinum spiral coil is used as the counterelectrode. After etching, the surface of the wafer is rinsed with ethanol several times and dried under a dry nitrogen gas. The resulting porous films are removed from the bulk Si substrate by applying an electropolishing current density of 4 mA cm^−2^ for 3 min in an electrolyte solution of 3.3% HF in ethanol. The freestanding films are then placed in absolute ethanol and ultrasonically fractured, using an ultrasonic probe (Amp. 55%, 30 min, Vibracell 750 W, Sonics, USA), to produce particles ranging in size from 2 to 18 μm. All materials are supplied by Sigma Aldrich Chemicals, unless otherwise mentioned.

*Hydrosilylation of PSi microparticles*. Freshly etched PSi microparticles are chemically modified using thermal hydrosilylation of undecylenic acid (≥95%), using microwave irradiation (Intelowave MS-204WS LG), to form undecanoic acid-terminated (u-PSi) PSi. Briefly, 20 mg of microparticles are placed in an open Pyrex beaker containing 6 ml of undecylenic acid and are allowed to react for 6 min at 320 W. The resulting microparticles are thoroughly rinsed with acetone (Frutarom, Israel) and ethanol (99.9%, Merck) to remove unreacted species from the surface and then dried under a stream of nitrogen gas.

*Physical characterization of PSi microparticles*. High-resolution scanning electron microscopy is performed using a Carl Zeiss Ultra Plus Field Emission scanning electron microscopy, at an accelerating voltage of 1 keV. Particle size and size distribution are determined using confocal laser scanning microscopy (LSM 700, Carl Zeiss, Germany) and the image analysis software (Axio Vision Rel. 4.8 with auto-measurement module). Surface chemistry of the microparticles was characterized with ATR-FTIR spectroscopy using a Thermo 6700 FTIR instrument equipped with a Smart iTR diamond ATR device.

### Fluorescence labelling of porous Si microparticles

Twenty milligrams of undecylenic acid modified (u-PSi) microparticles are suspended in 2.5 ml of 0.2 M solution of N-(3-dimethylaminopropyl)-N_2_-ethylcarbodiimide hydrochloride (≥98.0%), and 2.5 ml of 0.1 M solution of N-Hydroxysulfosuccinimide sodium salt (Sulfo NHS, ≥98.0%) for 10 min. TRH solution (2.5 ml; Invitrogen, USA), consisting of 1.62 × 10^−3 ^M TRH in N,N-Dimethyl formamide (Frutarom), is added and the microparticles allowed to react under orbital shaking for 2 h at room temperature. Removal of unreacted TRH following labelling involved cycles of centrifugation (15,000 *g* for 5 min) and replacement of the supernatant with N,N-Dimethyl formamide and deionized water. This step is repeated several times till the red colour of TRH had disappeared, after which the microparticles are dried under a stream of nitrogen gas.

The *in vitro* effect of PBS with and without peroxynitrite on the fluorescent intensity of TRH-PSi microparticles (reported in Fig. 3) is studied by incubating batches of 2 mg of TRH-labelled particles in 2 ml of PBS or PBS supplemented with 2 mM SIN-1, under orbital agitation of 100 r.p.m. at 37 °C. At designated time intervals, each batch of microparticles is separated from the media by centrifugation and the microparticles are placed within black well and clear bottom 96 plates, after which their fluorescence is determined by high-throughput screening using IN Cell Analyzer 2000 (GE Healthcare). Fluorescence images of the labelled particles are captured using an excitation filter wavelength of 530–580 nm and an emission filter wavelength of 600–660 nm and acquired automatically with a × 10 objective. Area and the mean fluorescence intensity per pixel of each labelled microparticle and the number of microparticles are obtained using the IN Cell Developer software (GE Healthcare). Using this technique, every measurement captured the fluorescence of ~5,000 microparticles directly, eliminating any background signal that may arise from dye release to the media.

### *In vitro* degradation

*In vitro* degradation is followed by tracking TRH fluorescence intensity over time following the incubation of 2-mg TRH-labelled particles in 2 ml of PBS, under orbital agitation of 100 r.p.m. at 37 °C. At designated time intervals, aliquots are sampled and replaced with fresh PBS. TRH solutions are separated from the microparticles by centrifugation. Concentrations of the samples are determined by measuring the fluorescence intensity of the released dye using a microplate reader (Varioskan Flash; Thermo Scientific, Waltham, MA, USA) with an excitation of 585 nm. The peak intensity of TRH was found to be at 602 nm, and a calibration curve for the dye in PBS was used to determine the amount of dye released.

### The effect of ROS on Si erosion *in vitro*

To evaluate Si degradation kinetics, 2 mg of TRH-PSi microparticles were incubated in 2 ml of PBS or PBS supplemented with SIN-1 (2 mM), under orbital agitation of 100 r.p.m. at 37 °C. At designated time intervals, aliquots were sampled and replaced with fresh PBS or SIN-1 solution. The resulting liquid was stored at 4 °C for later analysis of total silicon by Varian Vista-Pro Inductively Coupled Plasma Atomic Emission Spectrometer (Varian Inc. Santa Clara, CA, USA; ICP-AES). The Si atomic emission peaks at 212.4, 251.6 and 288.2 nm are monitored. Si erosion is expressed as percentage of the total Si content of the studied PSi microparticles. Rate of erosion is characterized by an exponential decay model in the form of *m*_*Si*_(*t*)=*m*_∞_[1−*exp*(−*kt*)], where *m*_∞_ is the mass as t→∞ and *k* is a constant that characterizes the rate of erosion.^36^

### Breast cancer mouse model development and quantification of Si erosion

MDA-MB-231 Luciferase-expressing cells are used to induce breast cancer tumour following their culture in high-glucose DMEM medium (Lonza, USA) supplemented with 10% FBS (Gibco, USA), 1% PS/G (Lonza) and 0.7 mg ml^−1^ G418 (Sigma, USA) for positive selection. Upon reaching the required concentration, the cells are prepped for animal injection in HBSS (Lonza). Fifteen severe combined immunodeficiency female mice (Charles River, USA) were used (10 mice are induced with breast cancer and 5 serve as a healthy control), and the procedures approved by the Committee on Animal Care at the Massachusetts Institute of Technology. Each mouse is injected with 5 × 10^6^ cells in 50 μl, in the left mammary fat pad. Following tumour induction, mice are monitored for tumour development by imaging the Luciferase-expressing cells 15 min following intraperitoneal injection of luciferin solution using an *In Vivo* Imaging System (Perkin Elmer, USA). Tumour is also measured physically with a caliper. When the tumour reaches a volume of ~70 mm^3^, 2 mg of fluorescently labelled PSi microparticles in 30 μl of HBSS are injected intratumorally. Simultaneously, similar doses of particles are injected into healthy mammary tissues of five healthy mice. PSi erosion is monitored by tracking the fluorescent signal of labelled particles using the spectral unmixing protocol in order to subtract background and tissue autofluorescence (Ex filters: 500–570, Em filters: 600–660). Mice are imaged until tumour reached a volume of 1,000 mm^3^.

## Author contributions

E.S. and N.A. conceived the project; A.T.-B. fabricated, labelled and characterized PSi microparticles, performed all PSi *in vitro* degradation studies; Z.S. and M.B. performed animal work and the *in vivo* imaging; A.T.-B., E.S. and N.A. analysed the data and wrote the paper with input from all authors.

## Additional information

**How to cite this article:** Tzur-Balter, A. *et al*. Mechanism of erosion of nanostructured porous silicon drug carriers in neoplastic tissues. *Nat. Commun*. 6:6208 doi: 10.1038/ncomms7208 (2015).

## Supplementary Material

Supplementary Information Supplementary Figures 1-6 and Supplementary References.
