# Supplementary material for: Mechanism of erosion of nanostructured porous silicon drug carriers in neoplastic tissues
Source: Nat Commun. 2015 Feb 11;6:6208. doi: 10.1038/ncomms7208 (PMC4339882; doi:10.1038/ncomms7208)
Supplement: Supplementary Information — Supplementary Figures 1-6 and Supplementary References. [file ncomms7208-s1.pdf]

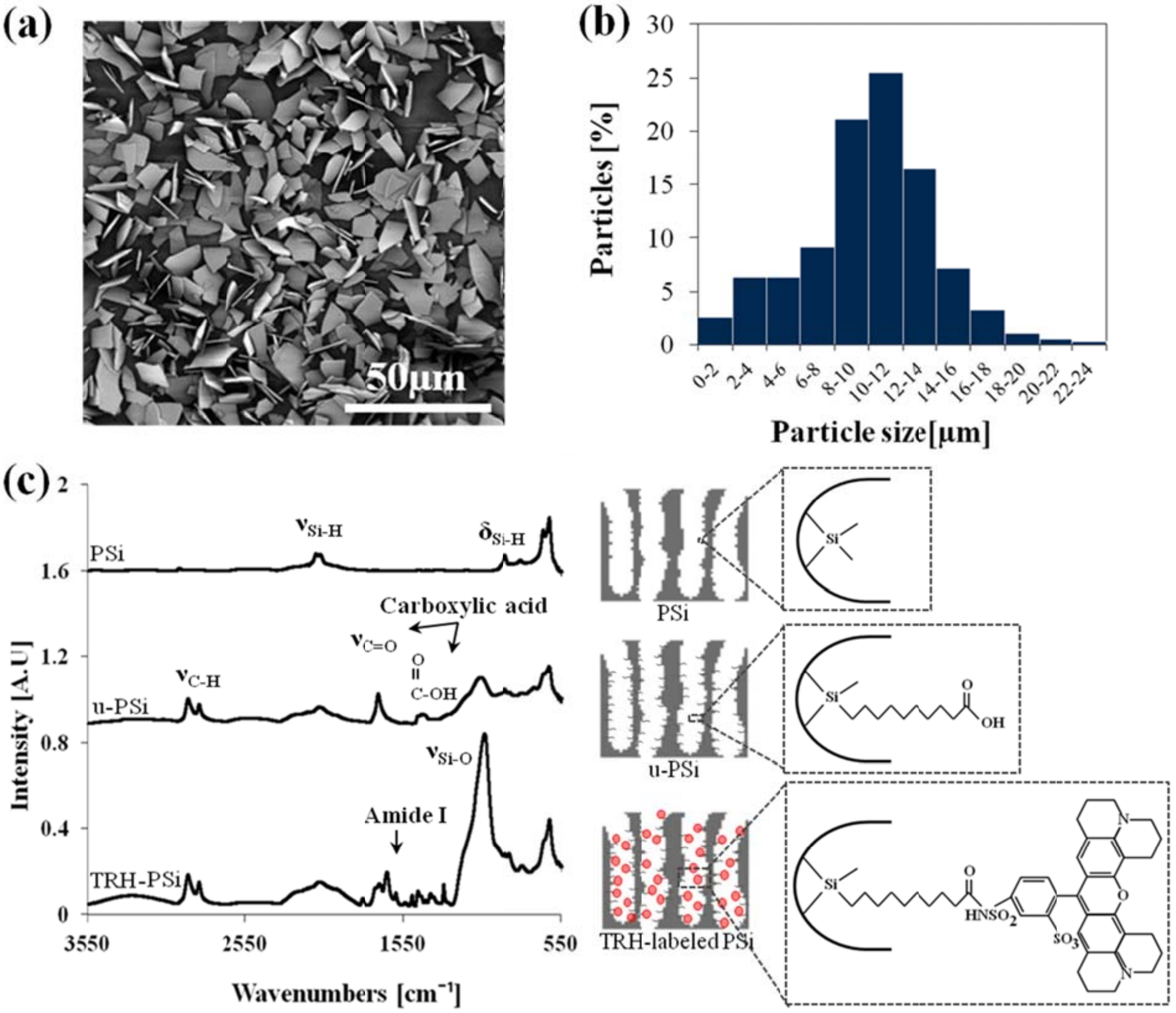

Supplementary Figure 1. Characterization of PSi microparticles. (a) High-resolution scanning electron micrograph of PSi microparticles, (b) Particle size distribution, and (c) ATR-FTIR spectra of PSi, before and following chemical modification and Texas-red labeling: freshly-etched PSi (PSi), undecanoic acid-terminated PSi (u-PSi), and Texas red-labeled PSi (TRH-PSi). The spectrum of PSi microparticles depicts bands characteristic of surface hydride species. Bands assigned to Si-H_x stretching modes (2085 cm^{-1}), and an additional band associated with Si-H deformations (907 cm^{-1}) are observed.[1] Following thermal hydrosilylation, the undecanoic acid-terminated PSi (u-PSi) displays bands characteristic of asymmetric and symmetric νCH_2 stretching vibrations at 2923 cm^{-1} and 2853 cm^{-1}, respectively. Furthermore, a strong band assigned to carboxylic acid νC=O stretching vibration at 1708 cm^{-1}, and a combination band due to the νC=O stretching and δO-H deformation vibrations of carboxylic acid at 1410 cm^{-1} are observed.[2,3] A small band at 1051 cm^{-1}, assigned to a ν(Si-O) stretching mode of surface oxide, is also observed, ascribed to a minor degree of substrate oxidation incurred during the hydrosilylation procedure. 1-Ethyl-3-[3-dimethylaminopropyl] carbodiimide hydrochloride (EDC) and N-hydroxysulfosuccinimide (Sulfo-NHS) were used to link the amines of the TRH molecules to the carboxylic acid end groups on the u-PSi scaffold, forming an amide bond. This is indeed confirmed by the ATR-FTIR spectrum of the TRH-PSi particles, displaying characteristics of amide I at 1654 cm^{-1} (C=O stretching vibrations). In addition, new bands at 1300 cm^{-1} (asymmetric νSO_2), 1382 cm^{-1} (asymmetric νSO_2), 1461 cm^{-1} (aromatic νC=C and νC=N), 1497 cm^{-1} (aromatic νC=C), 1541 cm^{-1} (aromatic νC=C), 1599 cm^{-1} (aromatic νC=C) and 1816 cm^{-1} (aromatic C-H out-of-plane deformation vibrations) are also recorded, ascribed to the characteristic groups of the fluorophore.

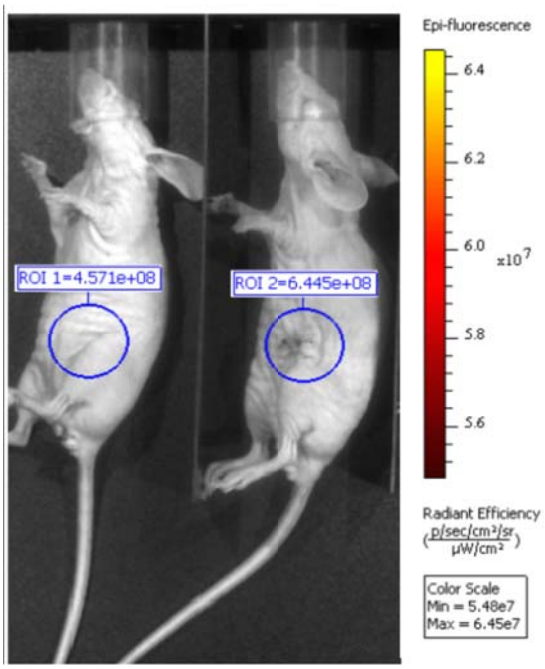

Supplementary Figure 2. The intrinsic luminescent signal following injection of PSi particles into the mammary fat pad of a SCID mouse, as measured using an In Vivo Imaging System (IVIS). Left: control mouse (mammary fat pad with no particles), Right: PSi microparticles injected. The luminescence signal of the injected microparticles is measured to be identical to the control mouse (mammary fat pad with no particles – only background signal).

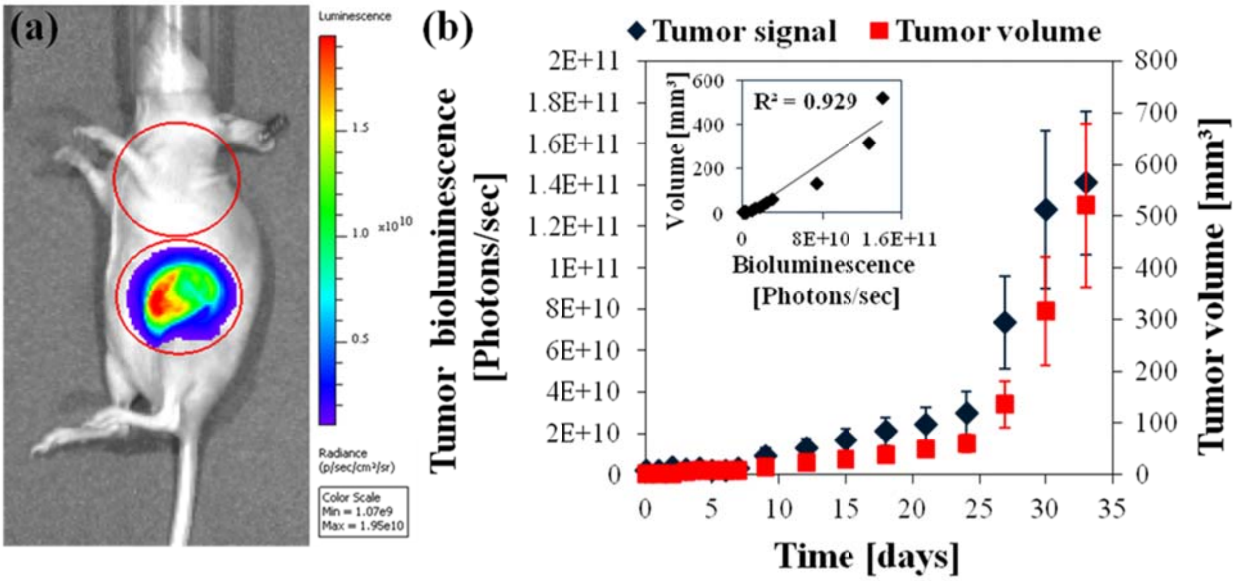

Supplementary Figure 3. Monitoring breast cancer tumor development. Breast cancer tumors were induced by injecting luciferase expressing human breast carcinoma cells (luc-MDA-MB-231) into the left mammary fat pad of SCID mice. These unique cells are engineered to express luciferase, thus enabling noninvasive imaging of tumor state by quantification of the omitted bioluminescent signal using an In Vivo Imaging System (IVIS). (a) Representative measurement of tumor bioluminescence using the IVIS technique, and (b) the bioluminescent signal and volume of breast cancer tumor induced in the mammary fat pad of 15 SCID mice. The results obtained by these methods exhibit a similar tumor growth behavior, displaying excellent linear correlation (R^2 = 0.929). The ability to correlate this bioluminescent signal with the physical volume of the tumor presents a major advantage and allows a continuous monitoring of tumor state, without the need to sacrifice animals at each time point.

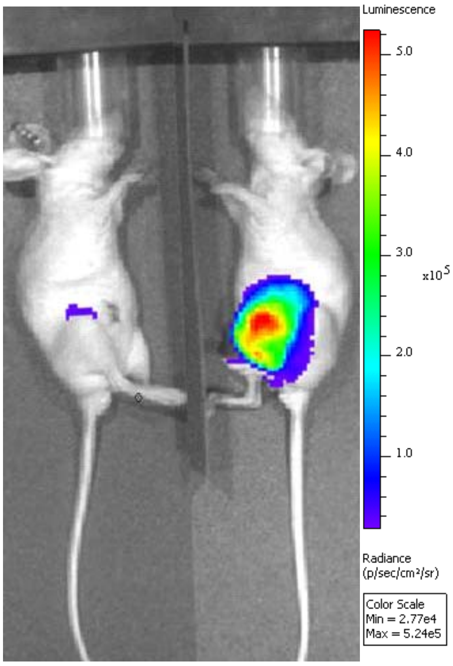

Supplementary Figure 4. Up-regulation of ROS in the tumor microenvironment. To directly demonstrate the *in vivo* up-regulation of ROS in the tumor microenvironment, we used an ROS-sensitive fluorophore to qualitatively assess the ROS levels in healthy and cancerous tissues. This fluorophore is optically silent unless a bond that is cleavable by oxygen reduces the quenching molecule attached to the dye. ROS fluorescence signal in healthy (left) and breast cancer bearing mouse (right) indicates on up-regulation of ROS concentration in the tumoral tissue.

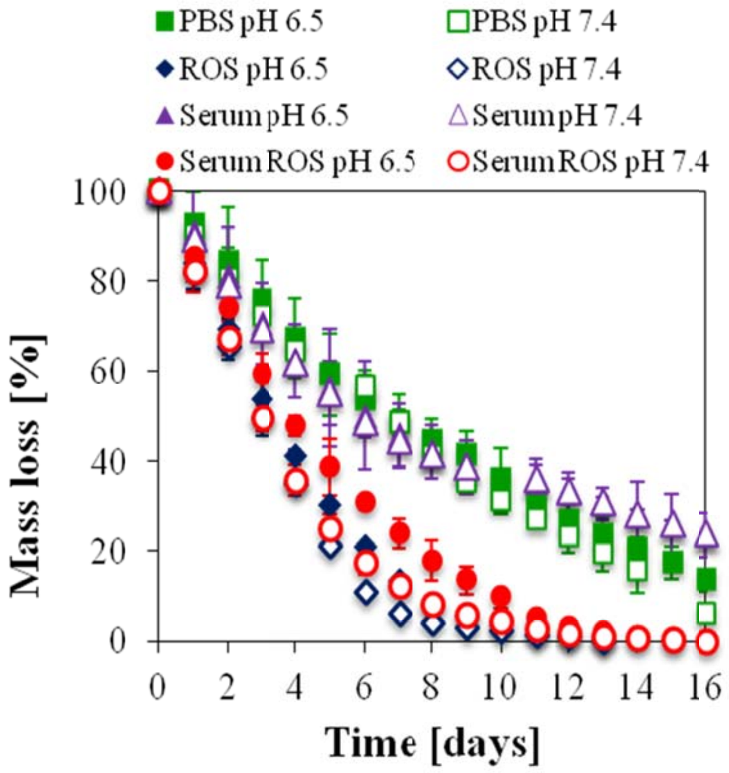

Supplementary Figure 5. The effect of pH, human serum, ROS and combinations thereof on the *in vitro* erosion process of PSi microparticles. The effects of acidic extracellular pH, protein adsorption, ROS and their combinations on the *in vitro* degradation of TRH-PSi microparticles were studied and compared. The results reveal that ROS is the most critical parameter that accelerates PSi degradation, as all degradation profiles carried out with ROS display a similar erosion behavior, while those carried out under the different conditions (PBS pH=7.4, PBS pH=6.5, Serum pH 7.4, Serum pH 6.5; no ROS) show a significantly slower degradation.

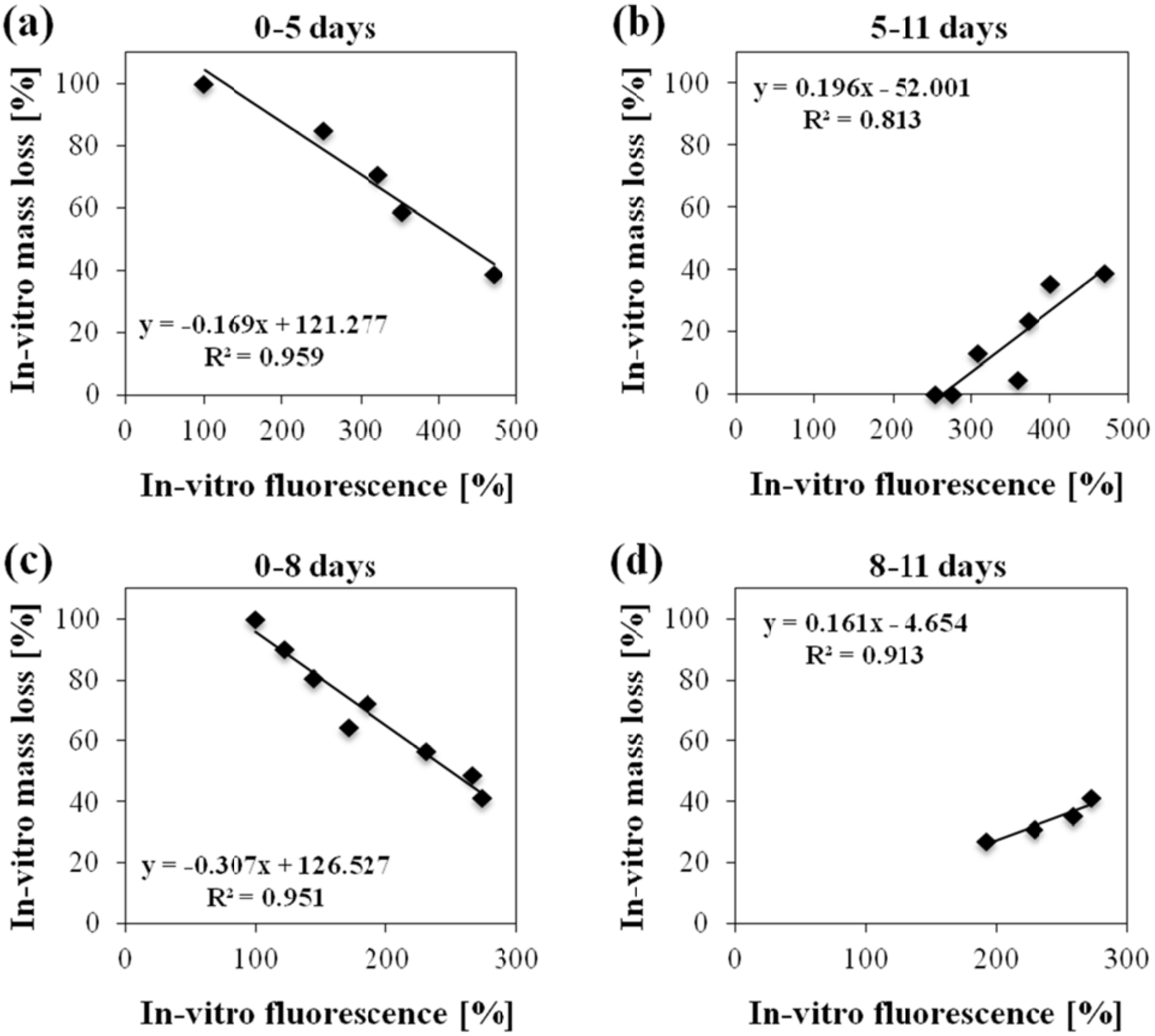

Supplementary Figure 6 Correlation between *in vitro* fluorescence intensity of TRH-labeled PSi microparticles and *in vitro* degradation in ROS and PBS buffers. (a) *In vitro* mass loss vs. *in vitro* fluorescence in ROS buffer at 0-5 days and (b) 5-11 days. (c) *In vitro* mass loss vs. *in vitro* fluorescence in PBS environment at 0-8 days and (d) 8-11 days.

Supplemental References

1 Anglin, E. J., Schwartz, M. P., Ng, V. P., Perelman, L. A. & Sailor, M. J. Engineering the chemistry and nanostructure of porous Silicon Fabry-Perot films for loading and release of a steroid. *Langmuir* **20**, 11264-11269, doi:10.1021/la048105t (2004).

2 Alvarez, S. D., Derfus, A. M., Schwartz, M. P., Bhatia, S. N. & Sailor, M. J. The compatibility of hepatocytes with chemically modified porous silicon with reference to in vitro biosensors. *Biomaterials* **30**, 26-34 (2009).

3 Schwartz, M. P., Cunin, F., Cheung, R. W. & Sailor, M. J. Chemical modification of silicon surfaces for biological applications. *Phys. Status Solidi A-Appl. Mat.* **202**, 1380-1384, doi:10.1002/pssa.200461106 (2005).
